# A New Hospital for Valparaiso

**Published:** 1895-12-28

**Authors:** 


### A NEW HOSPITAL FOR VALPARAISO.

### ST. AUGUSTINE.

The present general hospital of St. Augustine, Valparaiso, has, after the expression of a good many conflicting opinions on the part of the sanitary and medical authorities, been condemned as unsuitable for the purposes of a hospital, and it is proposed to erect another building more suitable to modern requirements at some distance from the old one.

One of the chief objections to the present hospital is its situation, which is practically in a valley. The new building, however, will stand in the Quinta Waddington, on the slope of one of the numerous granitic hills that surround the town, and will get the full benefit of the southern breezes prevailing in Valparaiso. The surrounding district is an eminently healthy one, and has always been singularly free from epidemics; the water supply is good and abundant.

To pass on to the building itself. The new hospital will contain 300 beds, of which nine, placed in a small building situated in the garden, are intended for convalescents, and forty-eight for maternity cases. These beds will be divided amongst the various blocks of which the hospital is composed, each block being separated from the next by a considerable space, traversed, however, by a covered gallery; and further being, except as regards kitchen and laundry arrangements, entirely independent of the rest of the hospital. The block will be further divided into an upper and a lower ward, communicating with each other by means of hydraulic lifts.

Ventilation is provided for by the erection of a central tower in each block to give egress to the ascending air; the strength of the current can be increased by heat. A central furnace is intended to secure a uniform temperature over the whole hospital. Besides the usual rooms and offices each block will contain a small ward to which the dying can be removed and tended apart from the other patients.

The utmost attention will be paid to the laundry arrangements. The soiled linen will be taken in a truck running upon rails straight from the wards to the disinfecting-room, from which it will pass into the aundry. The kitchen will be placed in the centre of the hospital, so as to communicate readily with all the different blocks, and to each ward will be allotted its own plates, dishes, and other utensils. Besides the kitchen proper there will be in the same block a dairy, pantry and scullery, a larder for meat, another for vegetables, and another for bread, and two rooms for the sisters in charge. Careful thought has also been given to the plans for the waiting-rooms, rooms for the reception of patients, bath-rooms, &c., in fact, the hospital is intended in every respect to fulfil modern requirements and to set an example in sanitary science to the other hospitals of Chili.

